# De novo transcriptome sequencing of *Rhododendron molle* and identification of genes involved in the biosynthesis of secondary metabolites

**DOI:** 10.1186/s12870-020-02586-y

**Published:** 2020-09-04

**Authors:** Guo-Lin Zhou, Ping Zhu

**Affiliations:** State Key Laboratory of Bioactive Substance and Function of Natural Medicines, NHC Key Laboratory of Biosynthesis of Natural Products, CAMS Key Laboratory of Enzyme and Biocatalysis of Natural Drugs, Institute of Materia Medica, Chinese Academy of Medical Sciences & Peking Union Medical College, 1 Xian Nong Tan Street, Beijing, 100050 China

**Keywords:** *Rhododendron molle* Transcriptome De novo assembly secondary metabolites biosynthesis

## Abstract

**Background:**

*Rhododendron molle* (Ericaceae) is a traditional Chinese medicinal plant, its flower and root have been widely used to treat rheumatism and relieve pain for thousands of years in China. Chemical studies have revealed that *R. molle* contains abundant secondary metabolites such as terpenoinds, flavonoids and lignans, some of which have exhibited various bioactivities including antioxidant, hypotension and analgesic activity. In spite of immense pharmaceutical importance, the mechanism underlying the biosynthesis of secondary metabolites remains unknown and the genomic information is unavailable.

**Results:**

To gain molecular insight into this plant, especially on the information of pharmaceutically important secondary metabolites including grayanane diterpenoids, we conducted deep transcriptome sequencing for *R. molle* flower and root using the Illumina Hiseq platform. In total, 100,603 unigenes were generated through de novo assembly with mean length of 778 bp, 57.1% of these unigenes were annotated in public databases and 17,906 of those unigenes showed significant match in the KEGG database. Unigenes involved in the biosynthesis of secondary metabolites were annotated, including the TPSs and CYPs that were potentially responsible for the biosynthesis of grayanoids. Moreover, 3376 transcription factors and 10,828 simple sequence repeats (SSRs) were also identified. Additionally, we further performed differential gene expression (DEG) analysis of the flower and root transcriptome libraries and identified numerous genes that were specifically expressed or up-regulated in flower.

**Conclusions:**

To the best of our knowledge, this is the first time to generate and thoroughly analyze the transcriptome data of both *R. molle* flower and root. This study provided an important genetic resource which will shed light on elucidating various secondary metabolite biosynthetic pathways in *R. molle*, especially for those with medicinal value and allow for drug development in this plant.

## Background

*Rhododendron molle* is a flowering perennial shrub indigenous to the south of the Yangtze River in China such as Guangdong and Guangxi Provinces. This plant grows mostly under shrubs or trees in the hilly area at the altitude of around 1000 m [1]. The medicinal properties of *R. molle* have been identified dating back to thousands of years ago [2, 3]. This plant has been recorded and depicted in the earliest Chinese medicinal book *Shennong Bencao Jing* (Shennong’s Classic of Materia Medica, compiled in the early period of the Eastern Han Dynasty dated from 25 AD to 220 AD). As one of the most famous traditional Chinese medicinal plant, *R. molle* has been extensively used as an anodyne and anesthetic since ancient times [4]. According to records in ancient medical books, both the dried flower and the root can be used as medicine to treat rheumatism, traumatic injury, migraine and neuropathic pain [1, 4]. In some rural areas, the plant was also be used as an insecticide [5]. Modern chemical studies have demonstrated that *R. molle* produces abundant secondary metabolites, mainly including terpenoids, flavonoids and lignans [6–9], among these compounds, the grayanane diterpenoids are the most important characteristic constituents in this plant, which account for the plant’s most significant bioactivities namely, analgesia, anti-nociceptive effects [7, 10], and hypotensive activity [11]. Pharmacological research proved grayanoids have significant analgesic activity for acute, inflammatory and neuropathic pain [7]. To date, many grayanoids have been isolated from flower and root of *R. molle* and the number is continually increasing. Flavonoids are common secondary metabolites with various pharmacological activities in natural plant, so far, numbers of flavonoids have been isolated from the flower of *R. molle*, such as quercetin, kaempferol, phloretin, vitexin [3, 12, 13]. Moreover, some interesting ligans have been isolated by Zhi et al. from the roots of *R. molle* [14]. In recent years, the RNA-Seq approach based on the NGS (next-generation sequencing) technology has been developed and widely used for fast and cost-effective transcriptome characterization of numerous vital medicinal plants like *Lilium regale* [15], *Glycyrrhiza uralensis* [16], *Eugenia uniflora* [17] and *Carthamus tinctorius* [18]. It also provides an effective way to accelerate discovering novel enzymes involved in the specific metabolic pathways [19, 20]. Recently, the RNA-Seq approach has also been used to analyze the flowering and flower color formation mechanism in *R. molle* [21]. The genes involved in the other secondary metabolic pathways remain unknown yet. In this study, deep de novo transcriptome sequencing for the flower and root of *R. molle* was performed using the Illumina Hiseq platform. A total of 100,603 unigenes with average length of 778 bp were obtained, among which 20,886 unigenes were over 1 kb in length. 57.1% of these unigenes were annotated based on sequence similarity searches and protein domain scanning against the publicly available databases. Moreover, bioinformatics analysis indicated that the genes encoding enzymes involved in the biosynthesis of the terpenoids backbone existed in the transcriptome of both *R. molle* flower and root. Total nine terpene synthases (TPS) were indentified including three diterpene synthases. In addition, the candidate genes putatively responsible for further backbone modifications were screened and a gene pool containing 61 cytochromes P450 (CYP) sequences was obtained. Putative members for biosynthesis of flavonoids and lignans were also identified in our transcriptome dataset. We profiled transcriptomes of flower and root separately and performed a comparative analysis to enrich the bioinformatics on the secondary metabolites. Our transcriptome data provided a valuable resource for the discovery of functional genes involved in various metabolic pathways, especially for the putative genes related to the biosynthetic pathways of the medicinally important secondary metabolites and will pave the way towards enhanced biosynthesis of secondary metabolites with medicinal properties through synthetic biology approach.

## Methods

### Materials and RNA extraction

*Rhododendron molle* was collected from Guangxi Institute of Botany in Guilin, Guangxi Province of China and was identified by professor Guang-Zhao Li of Guangxi Institute of Botany. A voucher specimen (ID-24757) was deposited in the herbarium at the Department of Medicinal Plants, Institute of Materia Medica, Chinese Academy of Medical Sciences (CAMS). The plant was grown in the greenhouse at the Institute of Medicinal Plant Development, CAMS. The roots and flowers were harvested and washed with tap water and ultrapure water successively, dried on filter paper. The roots were chopped into small pieces. All samples were frozen immediately in liquid nitrogen and preserved at − 80 °C before further processing. Total RNA was extracted from the root and flower using Trizol reagent and was treated with the RNase-free DNase I to eliminate genomic DNA. The quality and purity of the extracted RNA were assessed by spectrophotometer. The RNA integrity number (RIN) was checked by the Agilent Bioanalyzer 2100 system. Sample with RNA integrity number (RIN) value more than 8.0 was selected for further use.

### cDNA library construction and sequencing

One mircogram RNA sample was collected to construct the cDNA library using the NEBNext®Ultra™ RNA Library Prep Kit for Illumina® (NEB, USA) following manufacturer’s instructions. Briefly, the mRNA molecules were purified with Magnetic Oligo (dT) beads, fragmented and subjected to cDNA synthesis, then cDNA library was generated through PCR. The quality of each sample library was assessed using the Agilent Bioanalyzer 2100 system. Ultimately the transcriptome library per tissue was sequenced by Illumina HiSeq 2000 platform (Biomarker Technologies Corporation, Beijing, China) and the paired-end reads were generated.

### De novo transcriptome assembly and annotation

The raw reads obtained via the cDNA library sequencing were initially processed by trimming the adapter and low quality reads to produce clean reads. The clean reads were assembled using the Trinity software (version 2.5.1) to generate transcripts [22]. Then transcript analysis was performed to remove redundancies with TGICL software (version 2.1) and acquire unigenes without redundancy [23]. All assembled unigene sequences were subjected to similarity search against major public databases, including NCBI non-redundant protein (NR) (https://www.ncbi.nlm.nih.gov/protein/) [24], Swiss Prot database (https://www.uniprot.org/uniprot/) [25], Clusters of Orthologous Groups (COG) (http://www.ncbi.nlm.nih.gov/COG/) [26], eggNOG4 (http://eggnogdb.embl.de/) [27], Pfam (https://pfam.xfam.org) [28]. The blast algorithm was used to identify homologous sequences with a cut-off value less than 10^− 5^. The annotations of the best hits were recorded. Gene Ontology (GO) (http://www.geneontology.org/) was further used to categorize the function of the unigenes by Blast2GO software (version 2.5) with default parameters [29, 30]. The TransDecoder software (version 5.0.0) was used to predict the coding region sequence (CDS) and the corresponding amino acid sequence of unigenes, according to the alignment of amino acid sequence with protein domain sequence in the Pfam database.

### Functional characterization using KEGG

All the assembled unigenes were mapped against the Kyoto Encyclopedia of Genes and Genome (KEGG) database (http://www.genome.jp/kegg/) [31] using the BLASTX with the threshold *E*-value of < 10^− 5^. The KEGG orthology (KO) assignments were carried out via the KOBAS software (version 2.0) [32] with default parameters.

### Differential expression analysis

Gene expression levels were calculated by the fragments FPKM [33] (per kilobase per million fragments mapped) approach using RSEM software (version 1.2.19) [34]. The EBSeq software (version 1.6.0) was used to carry out differential expression analysis of two samples. Pvalue was adjusted using qvalue [35], the threshold of qvalue< 0.005 and log2 (fold change)| > 1 was set as the two criteria for significantly differential expression.

### Transcription factor analysis

To identify the transcription factor (TF) families, the assembled unigenes were inquired against the TF protein domains in the plant transcription factor database (PlnTFDB) by BLASTX [36] (plant transcription factor database) by BLASTX with an *E*-value cutoff 1E^− 06^.

### Identification of simple sequence repeats (SSRs)

For identification of SSR motifs, the unigenes generated from transcriptome sequences of both flower and root tissue were searched with MISA (Microsatellite searching Tool) (version 1.0). In this study, the microsatellites from mono-nucleotide to hexa-nucleotide were detected, and both the perfect (containing a single repeat motif) and compound repeats (containing two or more motifs separated by 100 base pairs) were identified.

### Phylogenetic analyses

The phylogenetic tree was constructed using the amino acid sequences of TPSs and CYPs from the *R. molle* transcriptome, as well as the representative functionally characterized proteins from other plant species. Accession numbers of protein sequences derived from GenBank and swissprot were listed in Additional file 1: Table S1. Amino acid sequences were aligned in ClustalX 2.1. The alignment was manually refined, removed the terminal gaps. The TPS and CYP phylogenetic trees were constructed using the MEGA7 software [37] by the neighbor-joining (NJ) method. The significance level for the phylogenetic tree was assessed by bootstrap testing with 1000 replicates.

### Identification of genes related to terpenoids

Custom databases of TPSs and CYPs were established according to the publicly available protein sequences. The sequences retrieved from GenBank are given in Additional file 2: Table S2. The tBLASTn program was conducted to mine the candidate genes from the generated assemblies, with the *E*-value threshold of 1.0 × 10^− 50^ and the minimum read length of 500 base pairs. All the identified unigenes were validated by using BLAST search in NCBI database.

### Real-time PCR

RNA samples were isolated from the roots and flowers tissues. Reverse transcription was performed using the TransScript® One-Step gDNA Removal and cDNA Synthesis Super Mix kit (TransGene, Beijing, China) following manufacturer’s instructions. The reaction was carried out at 42 °C for 15 min and 80 °C for 5 s. UltraSYBR Mixture (CWBIO, Beijing, China) and LightCycler480 II (Roche, Switzerland) Real-Time PCR System were used to conduct real-time quantification. The reaction mixture (20 μL) contained 10 μL of 2 × UltraSYBR Mixture, 0.5 μL of each forward and reverse primers, and 1 μL (150 ng/ul) of template cDNA. The PCR amplification procedure was as follow: 95 °C for 10 min and 40 cycles of 95 °C for 15 s, 60 °C for 1 min. The gene-specific primers were designed using Primer 5.0 software and were listed in Additional file 3: Table S3. The GAPDH gene was used as an internal standard, Ct values were determined based on three biological replicates of each sample and calculated using the 2^–△△Ct^ relative quantitative method [38].

## Results

### Transcriptome sequencing, and de novo assembly

Two cDNA libraries were constructed from the total RNA of *R. molle* flower and root, respectively. The libraries were sequenced using the Illumina HiSeq 2000 platform and approximately 26.58 Gb of clean data (89 million reads) were generated. The quality check showed that the base quality was above Q30 for 92% reads, and raw reads were trimmed prior to assembly. The adapter and low quality reads were trimmed, and the short reads (< 50 bp) were also removed. Then, 47,559,180 and 41,387,924 high quality reads were obtained from the flowers and root libraries, respectively, for further analysis. Trinity software and TGI clustering tool (TGICL) were used for the de novo assembly and removed redundant clusters, a total of 100,603 unigenes were generated, with average length of 778 bp, the N50 length of 1384 bp, and the GC content of 47.7%. 40.88% (41,129) of the assembled unigenes were longer than 500 bp, and 20.96% (20,886) longer than 1000 bp. The length of the most unigenes fell between 200 bp and 2000 bp, as shown in Additional file 4: Figure S1a. Additionally, a total of 76,198 coding sequences (CDS) with average length 515 bp were predicted, including 20,213 (26.5%) complete CDSs. Among all predicted CDSs 18,106 (23.8%) were longer than 200 bp in length (Additional file 4: Figure S1b). The highly qualified sequencing results would be in favor of subsequent functional annotations.

### Functional annotation

**Fig. 1:**
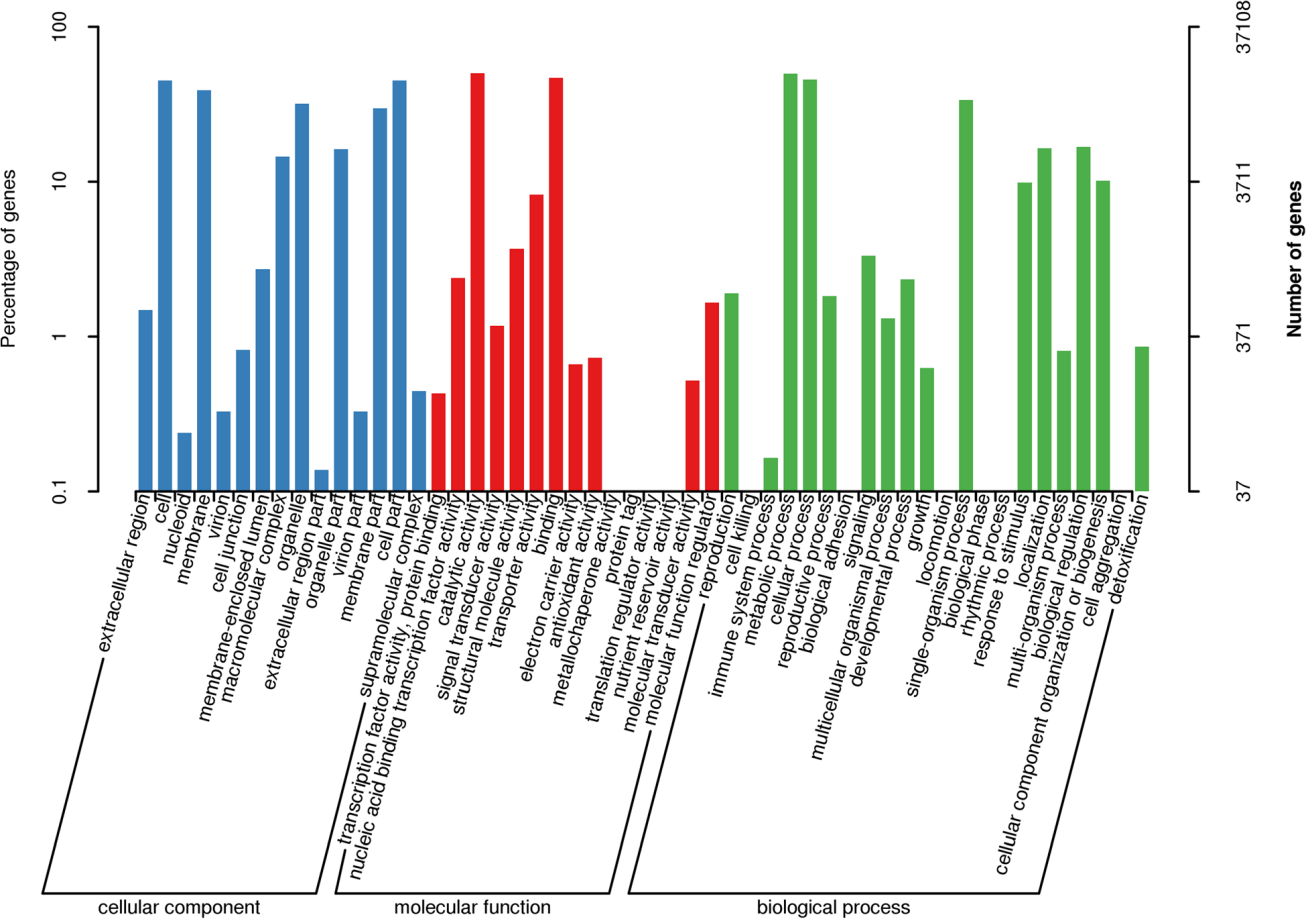
GO functional classifications of *R. molle* unigenes

For comprehensive annotation of assembled unigenes, sequence similarity search was performed against eight public databases. The result indicated that total 57,416 (57.1%) unigenes had significant matches in these public databases, while others were uninformative (e.g. “unknown” “unnamed” or “hypothetical protein”). Maximum annotation (56.2%) was resulted from NR database, while COG had the least number of annotated unigenes (16%). Additionally, 50.2, 36.9, 32.8, 27%, 26.8 and 17.8% unigenes acquired significant hits in the eggNOG, GO (Gene Ontology), Pfam (Protein family), KOG (euKaryotic Orthologous Groups), swissprot and KEGG, respectively (Table 1). Moreover, the *E*-value and identity distribution were calculated to further analyze the BLAST results. Statistical analysis revealed that 45.71% of the mapped sequences displayed apparent homology (< 1.0E^− 50^), while the remaining unigenes had the *E*-value ranging from 1.0 × 10^− 50^ to 1.0 × 10^− 11^ (Additional file 5: Figure S2a). In addition, the identity distribution showed that the majority of the mapped unigenes (73.64%) exhibited a similarity of > 60, 37.84% unigenes showed similarity between 60 and 80%, while 13.83% unigenes showed similarity between 50 and 60%, only 12.54% unigenes were < 50% (Additional file 5: Figure S2b). The higher identity along with high quality *E*-value proved reliability of the de novo assembly generated in this study. According to Nr annotation result, the top two species with the highest number of best hits were *Quercus suber* (8.88% matched unigenes) and *Vitis vinifera* (5.95% matched unigenes). (Additional file 5: Figure S2c). To facilitate the functional classification of the unigenes, GO annotation was conducted, which provided the ontology of defined terms representing gene product properties. GO annotations were further classified into three major classes as biological process, cellular component and molecular functions. A total of 37,108 sequences were identified based on sequence homology and can be classified into 52 functional groups (Fig. 1). Cellular components category was divided into 15 classes, in which the predominant groups corresponded to the cell (16,736 unigenes, 45.10%) and cell part (16,728 unigenes, 45.07%) followed by membrane (14,451 unigene, 38.9%), organelle (11,873 unigenes, 31.9%) and membrane part (11,098 unigenes, 29.9%). In the molecular function category, the top two groups were catalytic activity (18,589 unigenes, 50.09%) and binding (17,291 unigenes, 46.59%) which far outnumbered the unigenes corresponding to transporter activity (3066 unigenes, 8.26%) and structural molecular activity (1373 unigenes, 3.7%). In the 22 groups of biological process, the most abundant unigenes belonged to metabolic processes (18,425 unigenes, 49.65%), indicating rich secondary metabolites accumulated in *R. molle*, followed by those taking part in cellular processes (17,024 unigenes,48.65%) and single-organism process (12,502 unigenes, 32.73%).

**Table 1:** Annotation of unigenes against eight different databases

Annotated database	Annotated number	Percentage of annotated genes (%)
Nr	56,529	56.2%
eggNOG	50,523	50.2%
GO	37,108	36.9%
Pfam	32,970	32.8%
KOG	27,211	27%
Swissprot	26,983	26.8%
KEGG	17,906	17.8%
COG	16,102	16%
**All annotated**	**57,416**	**57.1%**

### Pathway analysis by Kyoto Encyclopaedia of genes and genomes (KEGG)

**Fig. 2:**
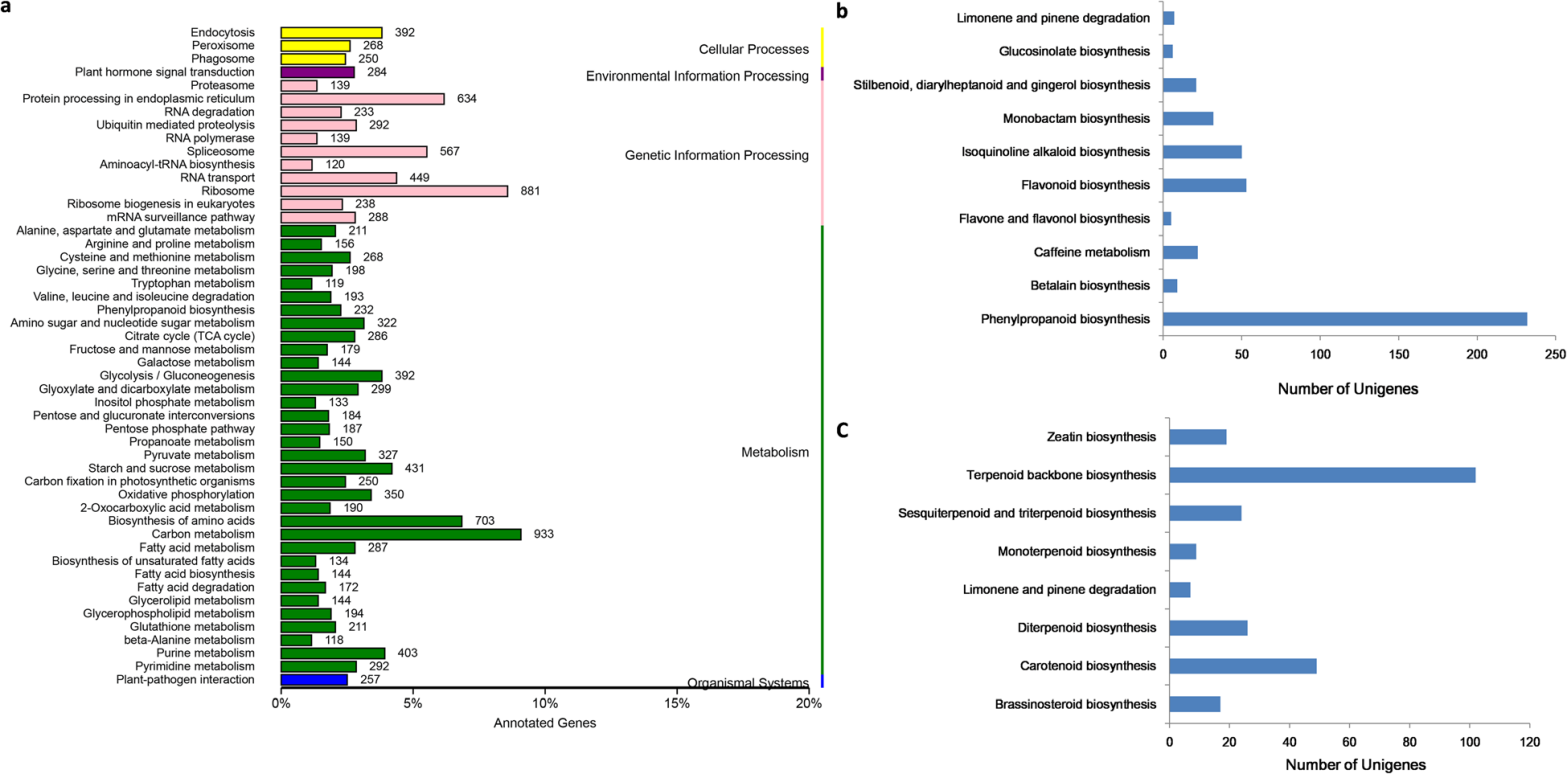
KEGG annotation of *R. molle* unigenes. **a** KEGG functional classifications of assembled unigenes*.* **b** Classifications of the subcategory “biosynthesis of other secondary metabolites”. **c** Classifications of the subcategory “ metabolism of terpenoids and polyketides “

Genes within the same pathway usually cooperate with each other to exercise their biological functions. Pathway-based analysis aid in understanding those functions and identification of unigenes involved in various biosynthetic pathways. In this study, KEGG pathway analysis was performed with the threshold *E*-value of < 10^− 5^. A total of 17,906 (17.8%) unigenes were significantly matched into the KEGG database which were divided into five primary categories, including cellular process, environmental information processing, genetic information processing, metabolism, and organismal systems comprising 130 pathways (Fig. 2a). In our dataset, the highest numbers of unigenes were grouped into “carbohydrate metabolism (993 unigenes)” followed by “ribosome (881 unigenes)” and “biosynthesis of amino acid (703 unigenes)”. We further explore the unigenes related to secondary metabolism, a total of 11 pathways including 437 unigenes were found to participate in “biosynthesis of other secondary metabolites”, among which the most unigenes were enriched in the beta-Alanine metabolism (118 unigenes) (Fig. 2b), followed by‘Phenylalanine biosynthesis’ (100 unigenes) and ‘flavonoid biosynthesis (53 unigenes). Furthermore, “metabolism of terpenoids and polyketides” subcategory contained 8 pathways including 253 unigenes, the cluster for ‘terpenoid backbone biosynthesis’ representing the largest group (102 unigenes), followed by carotenoid biosynthesis (49 unigenes) and diterpenoid biosynthesis (26 unigenes) (Fig. 2c).

### Over view of differentially expressed genes

**Fig. 3:**
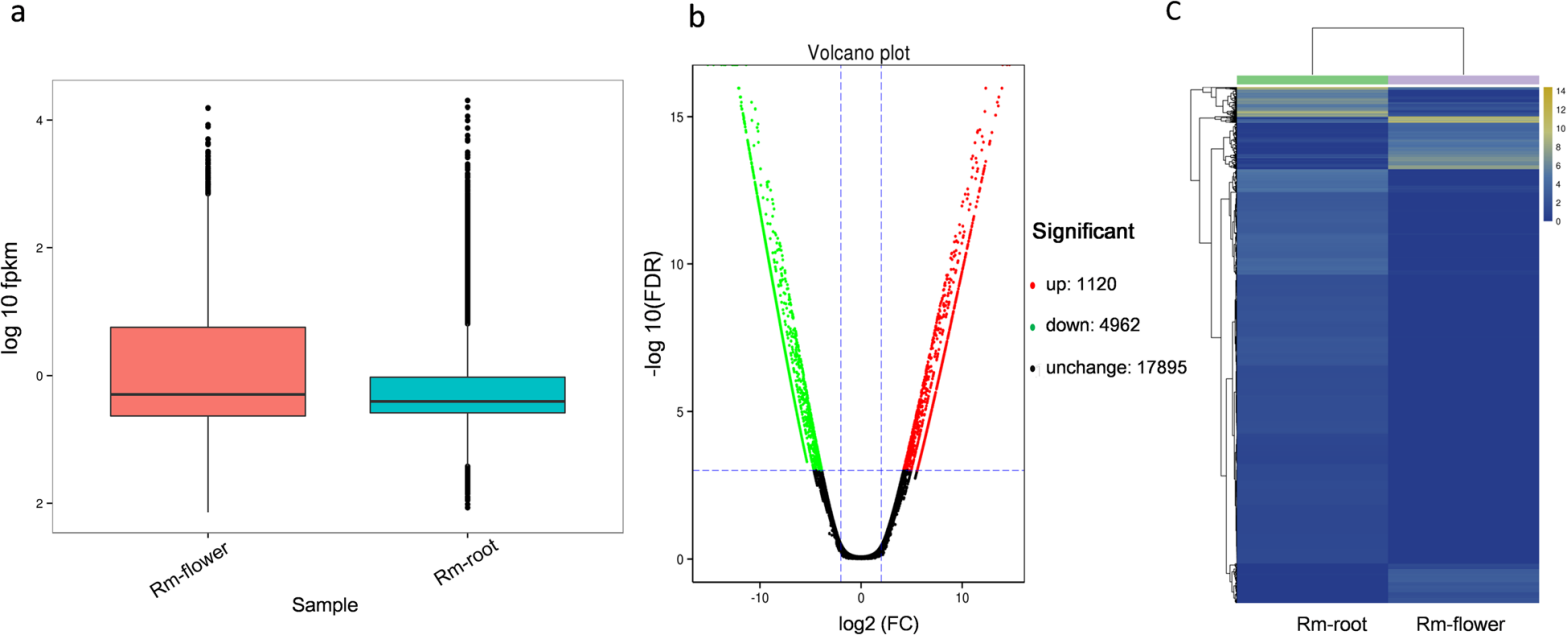
Unigene expression profiles in flower and root of *R. molle*. **a** Boxplot of FPKM value of root and flower samples. **b** Volcano plots of differential expressed genes between flower and root. **c**. Heat-map showing cluster analysis of differentially expressed genes between flower and root. The colour key is normalized by the log2 (FPKM) expression values, yellow and blue represent increased and decreased expression, respectively

**Fig. 4:**
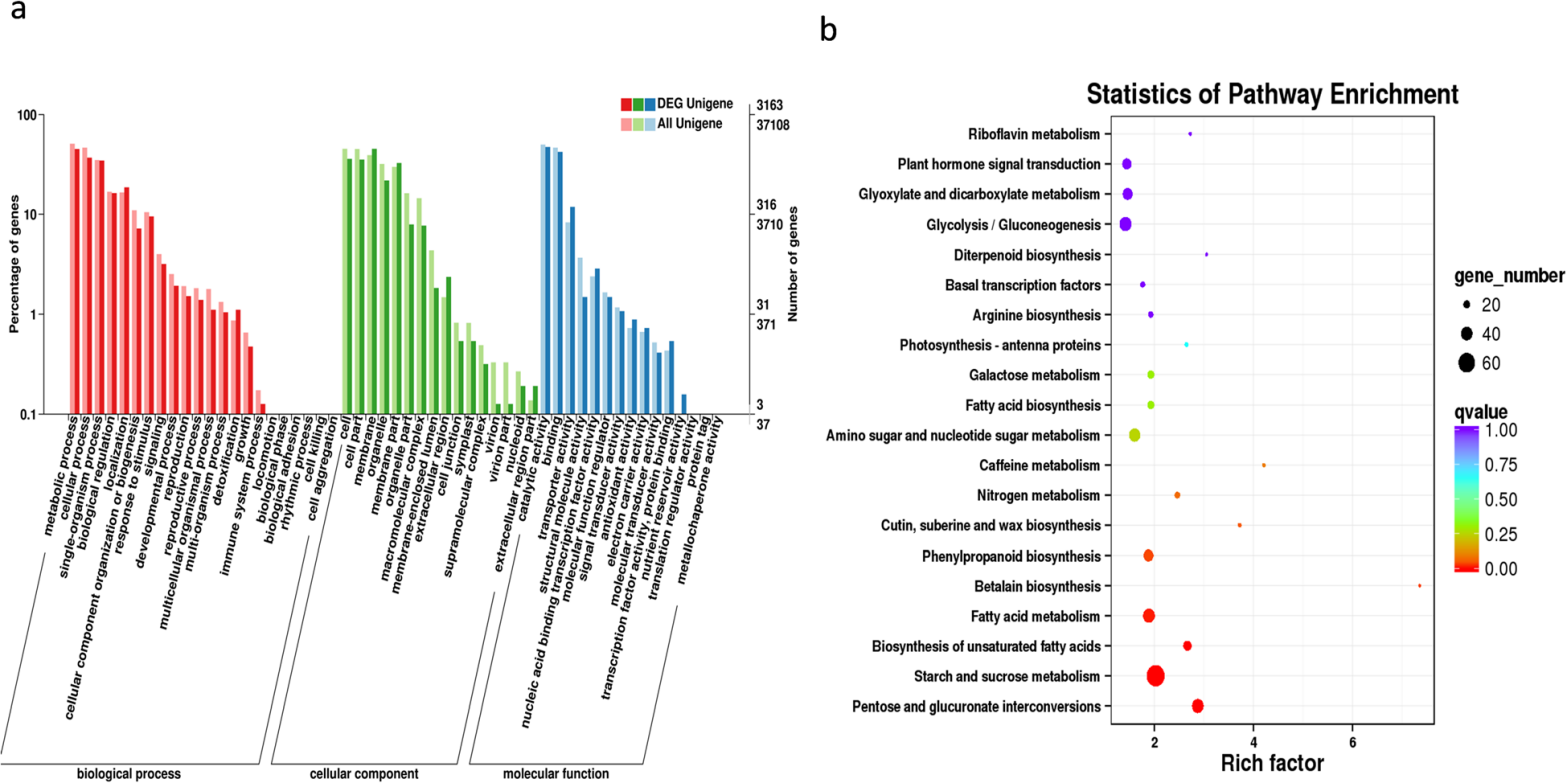
GO and pathway enrichment analysis of DEGs. **a** The gene enrichment of each secondary GO terms under the background of differentially expressed unigenes and all unigenes. **b** Each circle represents a KEGG pathway, the size of the circle is proportional to the number of unigenes enriched in the pathway. The colour of the circle represents qvalue, the smaller qvalue indicates the more reliable for the significance of differential expression genes in this pathway

We performed DEGs analysis of the two transcriptome libraries to discover the unigenes with significant difference in expression. FPKM value was used to measure unigenes expression levels. The overall expression levels of flower unigenes were higher than root unigenes (Fig. 3a). Further analysis revealed that out of 100,603 unigenes generated from the combined assembly of both flower and root transcriptomes, 6082 unigenes were differentially expressed in flower and root, including 1120 up-regulated and 4962 down-regulated unigenes in root vs flower (Fig. 3b), among which 507 unigenes were expressed uniquely in flower. Hierarchical clustering of the 6082 DEGs showed that the two tissues clustered relatively tight (Fig. 3c), indicating that some DEGs may involved in the same metabolic pathway. Out of 6082 unigenes, 5314 were annotated using different databases. For the GO enrichment analysis, the flower-specific up-regulated unigenes were assigned to several ontologies based on sequence homology, including 1254 for cellular component, 716 for biological process, and 1133 for molecular function. In the biological process category, the GO terms ‘metabolic process’ (GO:0008152) was most significantly enriched (304 unigenes), indicating the presence of vital metabolic activities in flowers (Fig. 4a). To further understand the involved metabolism pathways of DEGs, the KEGG enrichment analysis was performed. A total of 1433 unigenes referring to 115 KEGG pathways were identified, the top three most abundant DEGs enrichment pathways were carbon metabolism (78 unigenes, 5.4%), starch and sucrose metabolism (66 unigenes, 4.6%), and biosynthesis of amino acids (61 unigenes, 4.3%), all of them were related to primary metabolism (Fig. 4b). Besides, DEGs were also enriched in biosynthetic pathways of secondary metabolites, a total of 222 DEGs involving 20 biosynthetic pathways were identified, among them 32 DEGs and 6 DEGs were clustered in flavonoid and phenylpropanoid biosynthetic pathways respectively. Moreover the “metabolism of terpenoids and polyketides” subcategory contained 8 pathways including 27 DEGs, and the highest numbers of DEGs (7) were clustered into terpenoid backbone biosynthesis. Furthermore, one DEG in monoterpenoid biosynthesis, four DEGs in sesquiterpenoid and triterpenoid biosynthesis, and six DEGs in diterpenoid biosynthesis (Additional file 6: Table S4) Further research on these genes can offer an improved understanding of terpenoid biosynthetic pathway.

### Analysis of the secondary metabolic pathways

#### Identification of genes involved in terpenoid backbone biosynthesis

**Fig. 5:**
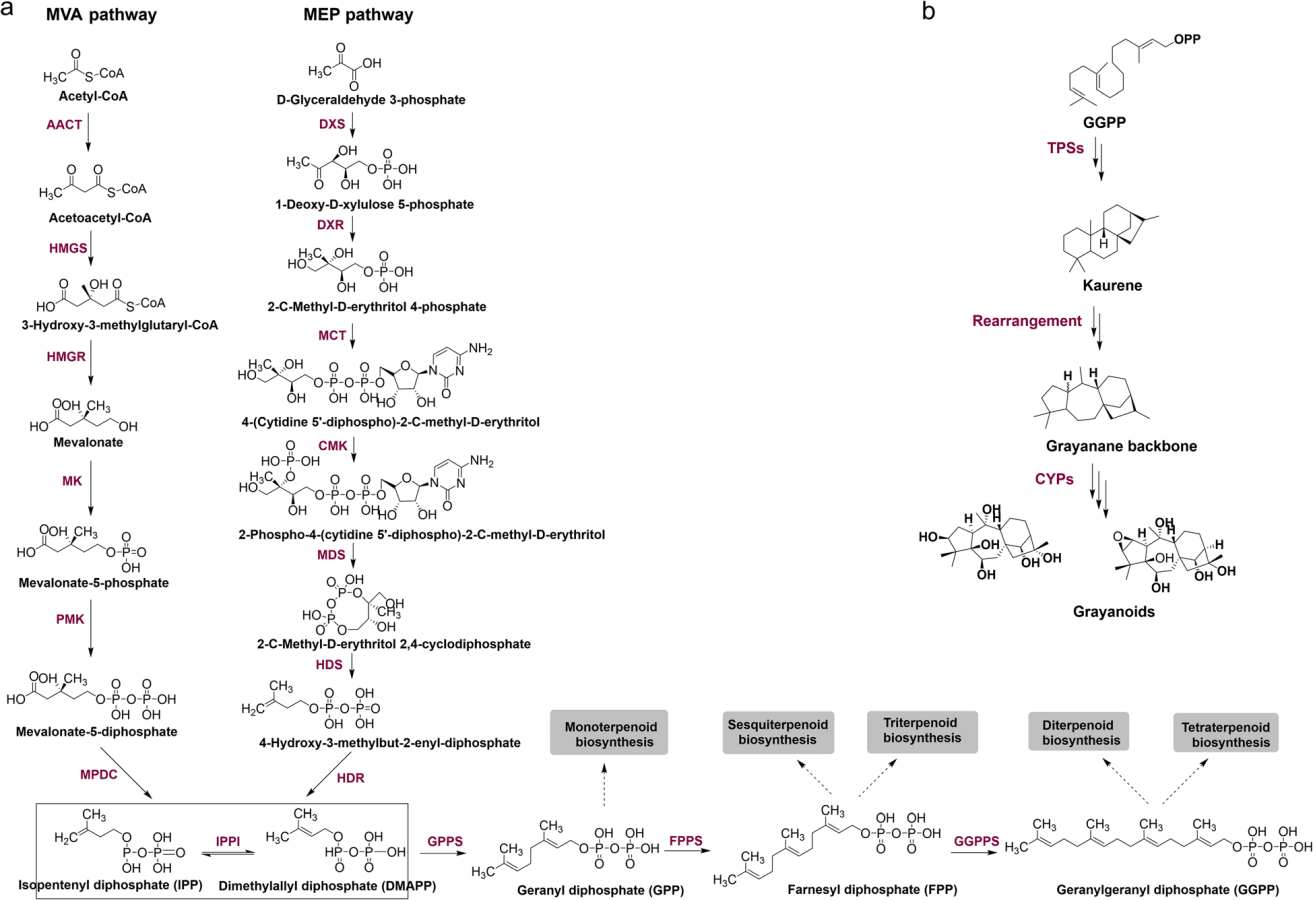
The terpenoid biosynthetic pathway in *R. molle*. **a** The biosynthetic pathway of terpenoid backbone. **b** The proposed biosynthetic pathway of grayanane diterpenoids

Terpenoids are the major secondary metabolites accumulated in *R.molle*, especially grayanoids which belong to the tetracyclic diterpenoid. The biosynthesis process of terpenoids can be divided into two stages, namely, the synthesis of terpenoid backbone and specific terpene formation and modification. The terpenoid backbone is synthesized from dimethylallyl diphosphate (DMAPP) and isopentenyl diphosphate (IPP), the general C-5 building blocks [39, 40]. In plants both the cytosolic mevalonate (MVA) and the plastids methylerythritol phosphate (MEP) pathway contribute to supplying DMAPP and IPP with cross flow [41, 42]. DMAPP was then sequentially condensed with IPP catalyzed by prenyltransferase leading to the formation of the starting precursors of different classes of terpenes, i.e., geranyl diphosphate (GPP, C-10) for monoterpenes, farnesyl diphosphate (FPP, C-15) for sesquiterpene and geranylgeranyl diphosphate (GGPP, C-20) for diterpenes [39] (Fig. 5a). Based on the KEGG pathway assignment, a total of 102 unigenes for 17 key enzymes related to the biosynthesis of terpenoid backbone were annotated, accounting for 0.57% of all the assembled unigenes with pathway annotation. These unigenes were mainly distributed in the MVA (46 unigenes, 6 enzymes) and MEP (17 unigenes, 6 enzymes) pathways, which may participate in the biosynthesis of IPP, the common building block of terpenoinds. Moreover, several genes (28 unigenes, 3 enzymes) were distributed in the downstream. In most cases, more than one unigenes was annotated as the same enzyme, suggesting that these unigenes might represent different members of the same gene family or the different fragments of a single transcript. Corresponding unigenes were listed in Table 2. Among them, seven DEGs were discovered, three up-regulated unigenes were related to MEP pathway, including one for DXS, one for 2-C-Methyl-D-erythitol 2,4 cyclodiphosphate (MDS) and one for isoprene synthase. Besides, we also found three down-regulated unigenes which were involved in MVA pathway, including two for HMGS, one for PMK (Table 2). These results indicated that the MEP pathway was mainly responsible for synthesizing terpenoids in flowers.

**Table 2:** Unigenes involved in the terpenoid backbone biosynthesis in *R. molle*

Enzyme name	EC number	Unigene number	DEGs number
AACT	2.3.1.9	18	*
HMGS	2.3.3.10	10	2
HMGR	1.1.1.34	12	*
MK	2.7.1.36	1	*
PMK	2.7.4.2	2	1
MVD	4.1.1.33	3	*
DXS	2.2.1.7	3	1
DXR	1.1.1.267	1	*
CMS	2.7.7.60	1	*
MCS	4.6.1.12	1	1
HDS	1.17.7.1	2	*
HDR	1.17.7.2	2	*
IPPI	5.3.3.2	4	*
GPPS	2.5.1.1	12	*
FPPS	2.5.1.10	10	*
GGPS	2.5.1.1	6	*
ISPS	4.2.3.27	1	1
CHL P	1.3.1.83	3	1

* DEGs were not found AACT: acetyl-CoA acetyltransferase HMGS:hydroxymethylglutaryl-CoA synthase HMGR: hydroxymethylglutaryl-CoA reductase MK: mevalonate kinase PMK: phosphomevalonate kinase MVD: mevalonate diphosphate decarboxylase DXS: 1-deoxy-D-xylulose-5-phosphate synthase DXR: 1-deoxy-D-xylulose-5-phosphate reductoisomerase CMS: 2-C-methyl-D-erythritol 4-phosphate cytidylyltransferase MCS: 2-C-methyl-D-erythritol 2,4-cyclodiphosphate synthase

HDS: 4-hydroxy-3-methylbut-2-enyl diphosphate synthase HDR: 4-hydroxy-3-methylbut-2-enyl diphosphate reductase IPPI: isopentenyl diphosphate isomerase GPPS: Geranyl diphosphate synthase FPPS:Farnesyl diphosphate synthase GGPS: Geranylgeranyl diphosphate synthase ISPS:isoprene synthase CHL P:geranylgeranyl reductase

#### Enzymes involved in grayanoids biosynthesis

**Fig. 6:**
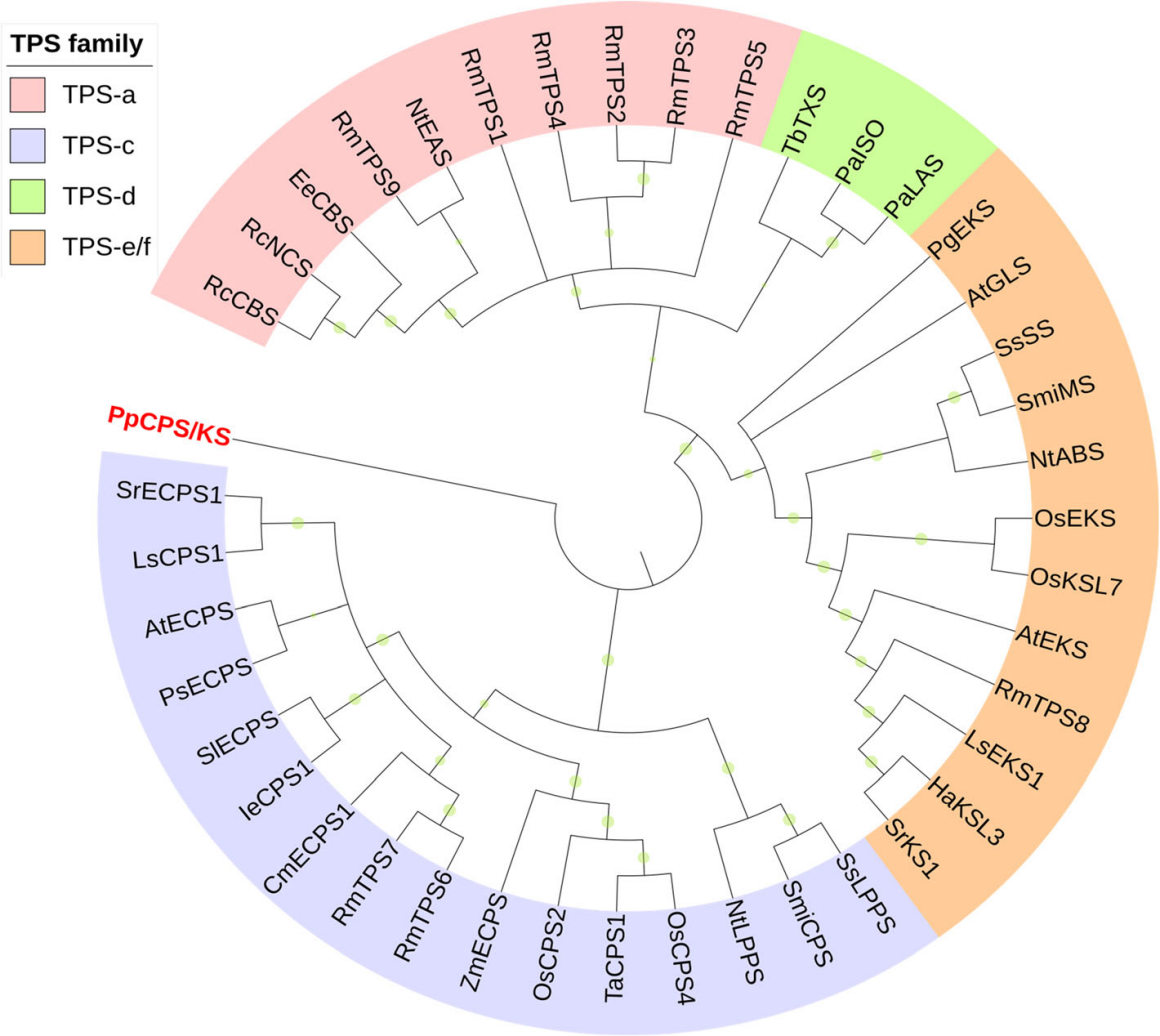
The phylogenetic tree of terpene synthases from *R.molle*. Neighbor joining tree. *P. patens* copalyl diphosphate synthase/kaurene synthase (PpCPS/KS) was used as the tree root

Previous investigations have reported that abundant grayanane diterpenes were isolated from the roots and flowers of *R. molle* [6, 7, 10] which are regarded as the characteristic metabolites of this plant and possess significant analgesic activity. The proposed biosynthetic pathway of grayanoids starts from the common precursor GGPP (20-carbon), which are converted to kaurene by terpene synthases (TPSs) firstly, then finally generate grayanoids. The biosynthesis process from kaurene to grayanane may involve oxidative rearrangement (Fig. 5b). The grayanane backbone undergoes modifications primarily through the activity of cytochromes P450 (CYP) enzymes. To identify TPS and CYP candidates, the custom databases were built based on the publicly available protein sequences, which represented the least populous sequence sets without redundancy. A panel of nine terpene synthases was identified from the transcriptome data according to the sequence homology to the NCBI NR database (Additional file 7: Table S5), in which three unigenes were annotated as linalool synthases involved in monoterpene biosynthesis, and one unigene as germacrene D synthase. Additionally, two copies of copalyl diphosphate synthase and one copy of ent-kaurene synthase were also identified, and details were shown in Table 3. These enzymes can be grouped into four families according to phylogenetic relationships (Fig. 6). Six out of these TPS candidates (RmTPS1–5, RmTPS9) belonged to the TPS-a family, and RmTPS8 was classified into the TPS-e/f family. All the above-mentioned TPSs possessed the features of a class I terpene synthase, and only two class II terpene synthase were discovered in our dataset, which were RmTPS6 and RmTPS7 belonging to the TPS-c family. In angiosperm, the formation of diterpene backbone requires both class I and class II terpene synthase, the specific functions of TPSs need to be further verified.

**Table 3:** Terpene synthase candidate genes of *R. molle*

Terpene synthase	Gene	Unigene ID	Annotation
**Mono-**	RmTPS3	c135826	linalool synthase
	RmTPS4	c61279	linalool synthase
	RmTPS5	c97331	linalool synthase
**Sesqui-**	RmTPS9	c71656	germacrene D synthase
**Di**	RmTPS6	c95076	copalyl diphosphate synthase
	RmTPS7	c86861	copalyl diphosphate synthase
	RmTPS8	c92044	ent-kaurene synthase
*	RmTPS1	c79171	putative terpene synthase 9 [*Quercus suber*]
*	RmTPS2	c91666	putative terpene synthase2 [*Camellia sinensis*]

**Fig. 7:**
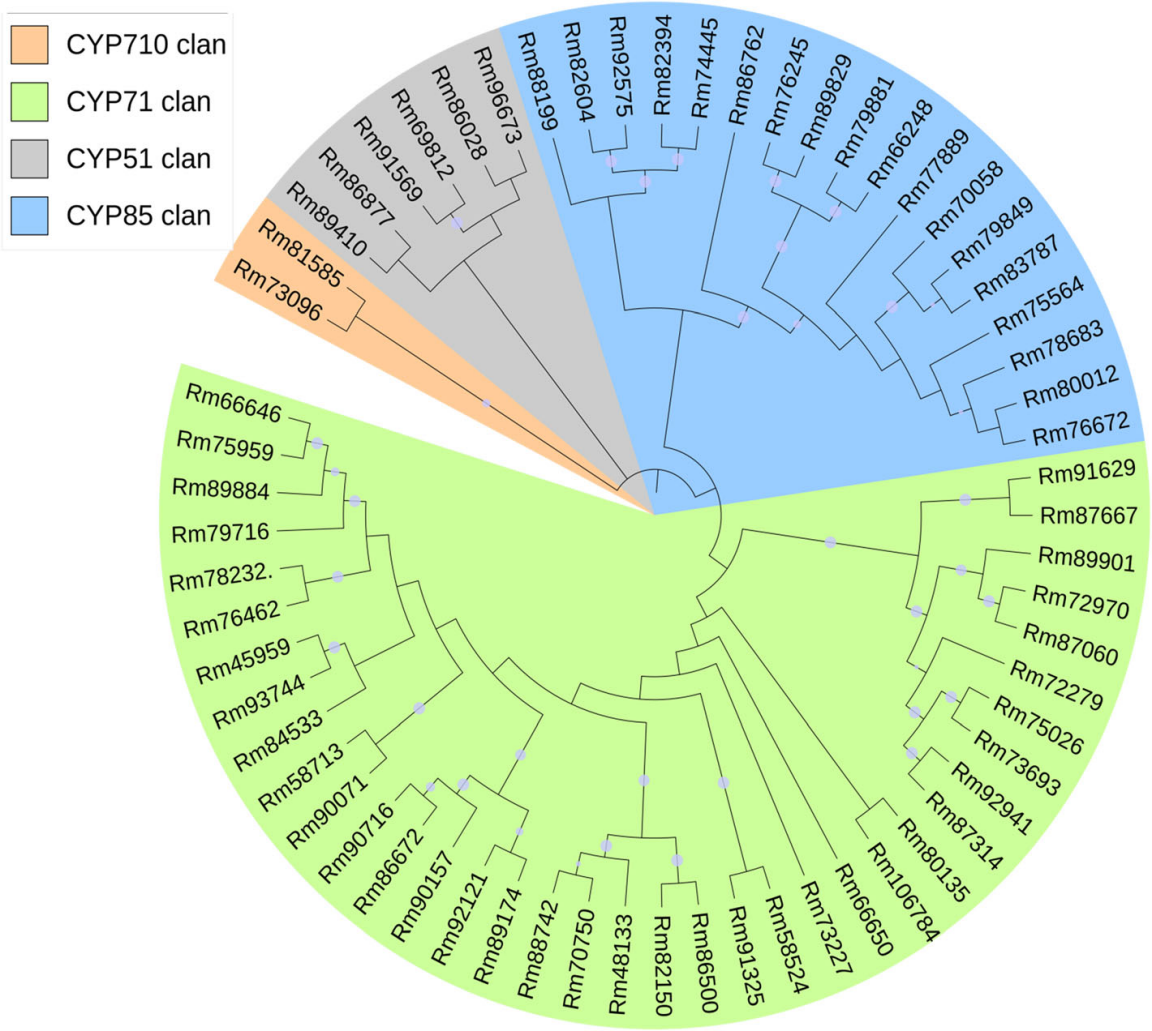
Phylogenetic analysis of CYPs from *R. molle*

**Fig. 8:**
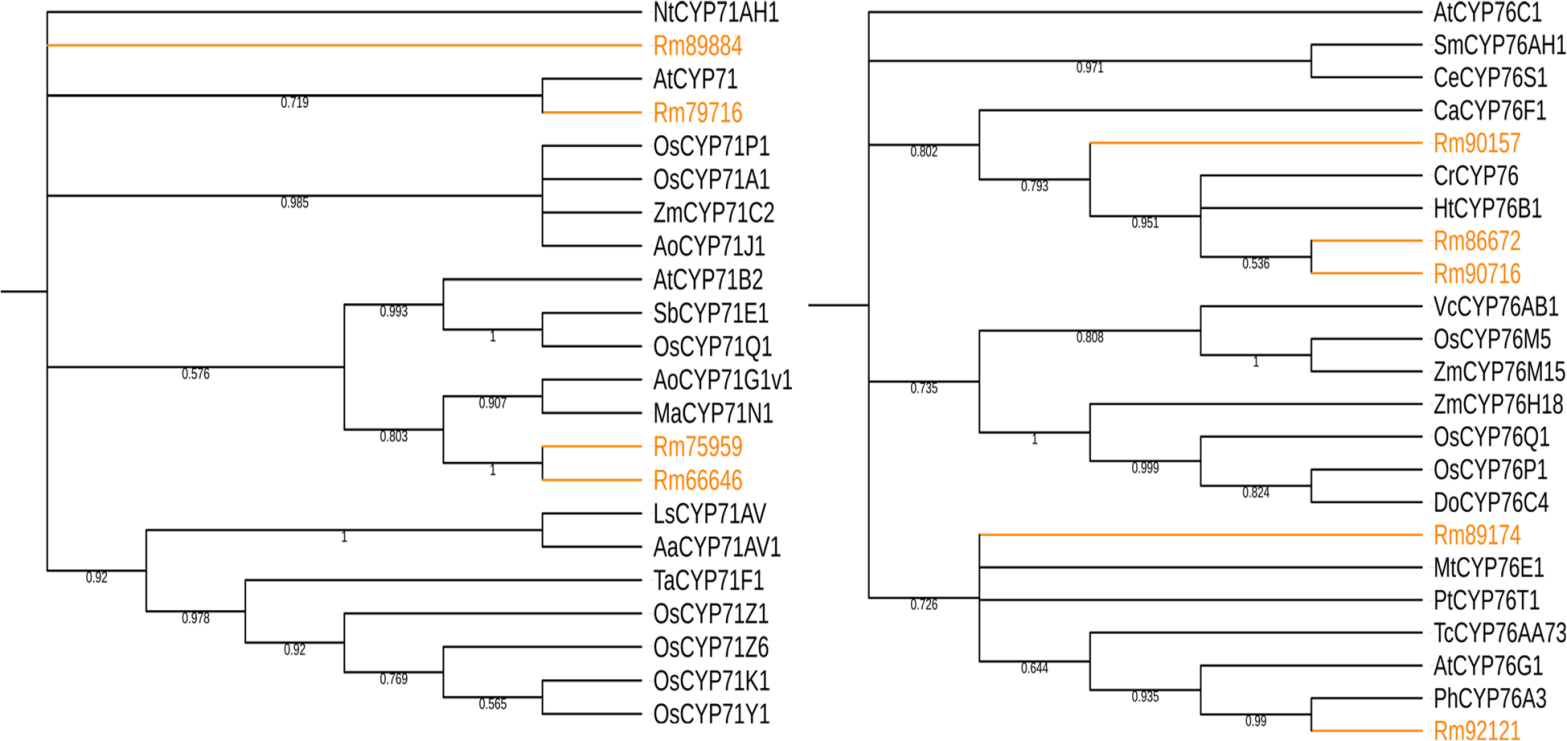
The phylogenetic analysis of CYP71 and CYP76 families in *R. molle*

**Fig. 9:**
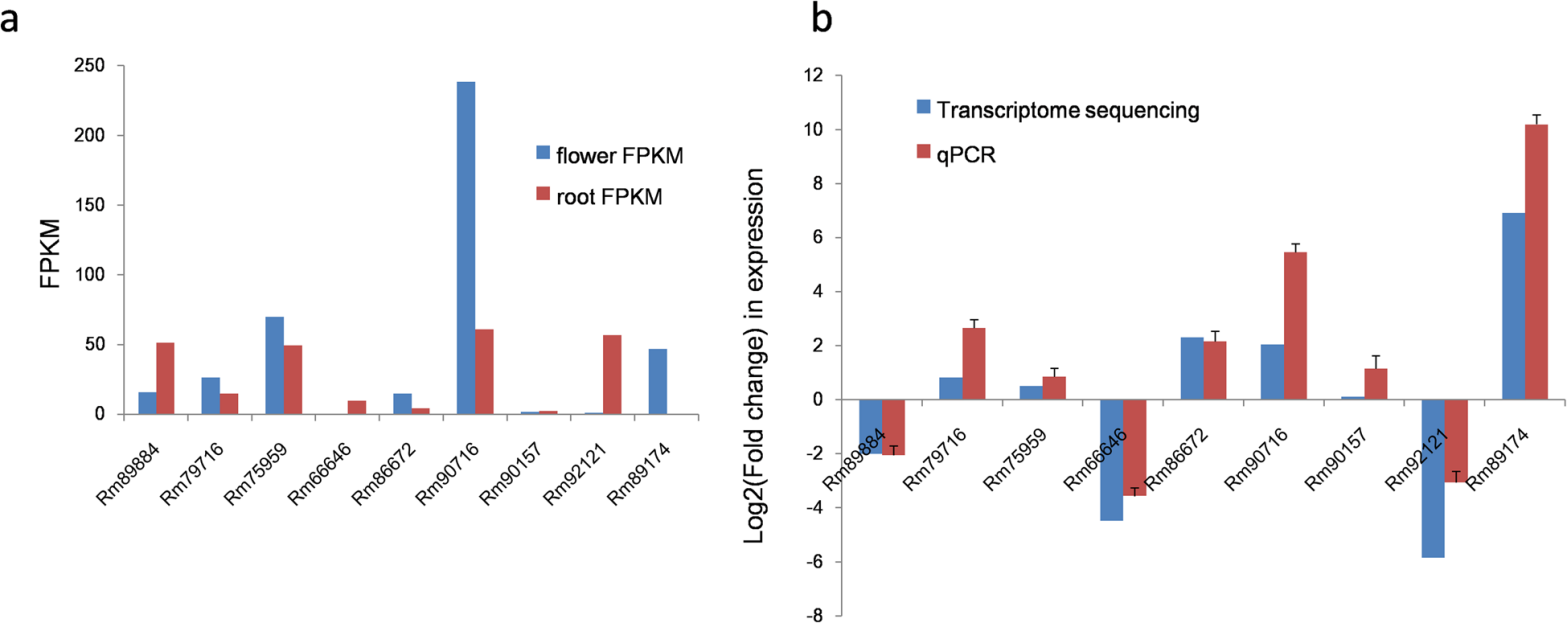
Relative expression of CYP71 and CYP76 families candidates in *R. molle*. **a** Expression profile of CYP candidates based on FPKM. **b** Gene expression levels of CYP candidates verified by qRT-PCR

Cytochrome P450 monooxygenases (CYP) represent the largest superfamily of enzymes (around 1% of the sequenced plant genomes) in plants, but only few CYPs involved in terpenoid metabolism have been characterized, making it challenging to identify CYPs in the specialized terpenoid biosynthetic pathway of *R. molle.* In this study, the *R.molle* flower and root transcriptomes were mined against a P450-specific protein database, and a total of 61 candidates were identified (Additional file 7: Table S5). Phylogenetic analysis classified these CYPs candidates into 4 clans, members of the CYP71 clan were the most represented (Fig. 7), this clan harbors the most of CYP families involved in plant secondary metabolism [43, 44]. Terpenoid metabolism in plants is dominated by a few CYP families, among which the CYP71 and CYP76 families are major contributor [44, 45]. We used reported proteins from these two CYP families as probes to investigate our transcriptomes and a total of nine CYP enzymes from CYP71 and CYP76 families were identified (Fig. 8). To further screen the highly probable CYP candidates, the gene expression levels were assessed based on the FPKM values. The results showed that most of the genes were expressed in both flowers and roots, but the expression levels were different. Specifically, Rm89174 was significantly up-regulated in the flower tissue, while Rm66646 and Rm92121 were highly expressed in the root tissue of *R. molle* (Fig. 9a). The expression levels of unigenes detected in FPKM analysis were further verified through qRT-PCR analysis (Fig. 9b). Generally, expression level measured by qRT-PCR was consistent with RNA-Seq data. The CYP unigenes showed accordant expressions in both qRT-PCR and FPKM analysis, confirming the reliability of the sequencing results. These results provide a reference for future functional characterization of TPS and CYP candidates involved in terpenoid biosynthesis in *R. molle*. Nevertheless, further research is warranted to uncover the true functions of these unigenes.

#### Lignan biosynthetic genes

**Fig. 10:**
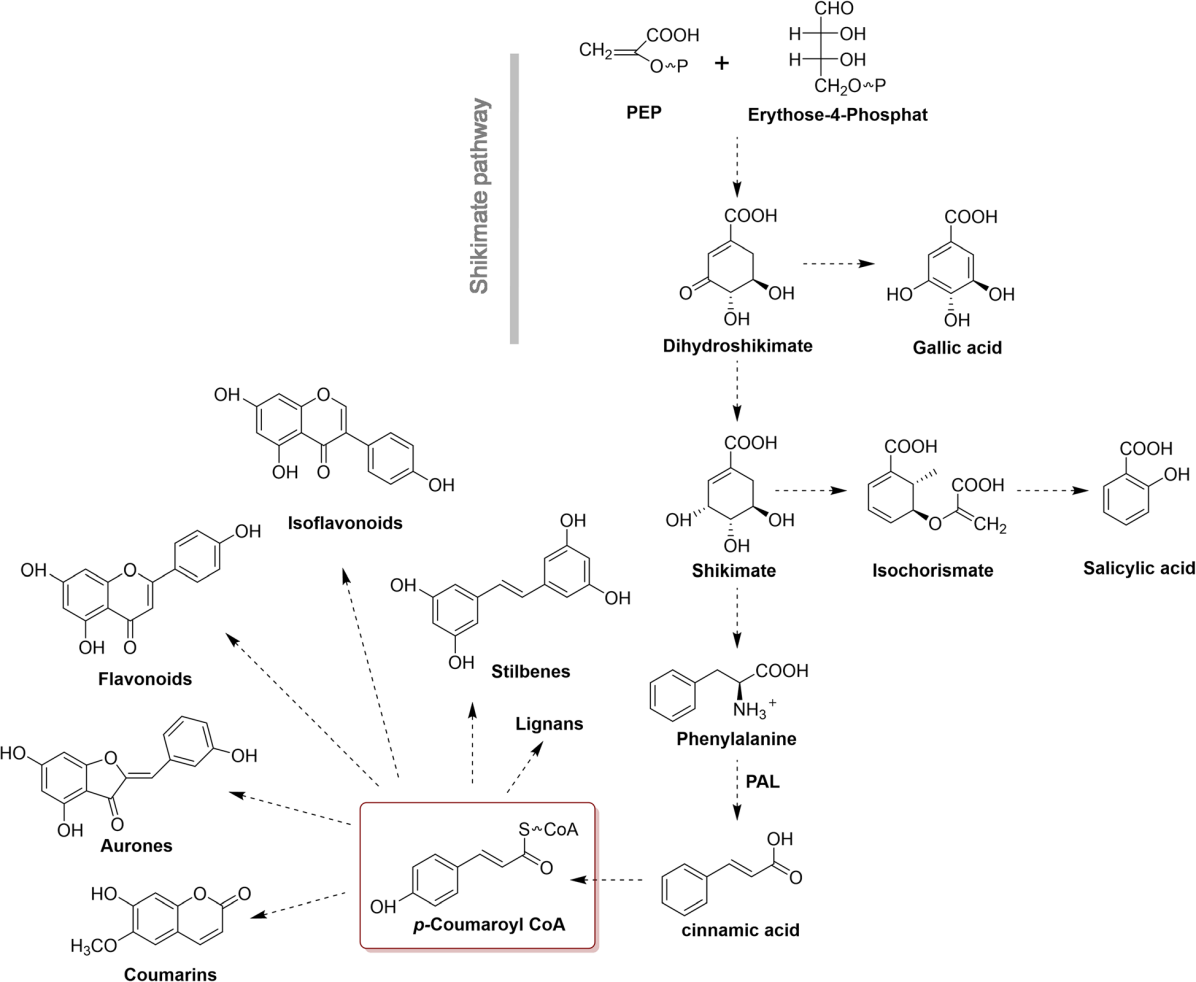
Phenylpropanoid Pathway and diversification of Phenylpropanoids in *R. molle*

Phenylpropanoids are derived from phenylalanine and comprise a large group of plant natural products with extensive bioactivities, such as hepatoprotection and antioxidation. These compounds are involved in all aspects of plant responses to both biotic and abiotic stimuli [46]. The general phenylpropanoid metabolism derives a large number of secondary metabolites using the few intermediates of the shikimate pathway as basic precursors. The biosynthetic pathway starts with the formation of cinnamic acid from phenylalanine, which results in the formation of cinnamoyl-CoA and p-coumaroyl -CoA. These CoA-activated compounds are the precursor for synthesizing lignans, flavonoids, flavonols as well as numerous other secondary metabolites (Fig. 10). In the present study, we performed KEGG analysis on both *R. molle* flower and root transcriptomes and the results revealed a total of 232 unigenes were involved in the phenylpropanoid biosynthetic pathway. Ten unigenes were annotated for coding phenylalanine ammonia-lyase (PAL) and three unigenes were annotated to code the trans-cinnamate 4-monooxygenase. Both of the two enzymes play a significant role in the formation of important intermediate cinamic acid. Besides, 18 unigenes were annotated as 4-coumarate-CoA ligase (4CL) and 6 unigenes were annotated to code cinnamoyl-CoA reductase (CCR). Moreover, enzymes at branching points were also identified, and the representative enzymes are listed in Table 4.

**Table 4:** Representative enzymes in phenylpropanoid biosynthetic pathway in *R. molle*

Enzyme name	Annotation	EC number	Unigene number
PAL	phenylalanine ammonia lyase	4.3.1.24	10
4CL	4-coumarate--CoA ligase	6.2.1.12	18
CCR	cinnamoyl-CoA reductase	1.2.1.44	6
CYP73A	trans-cinnamate 4-monooxygenase	1.14.14.91	3
HCT	shikimate O-hydroxycinnamoyl transferase	2.3.1.133	10
CYP98A	5-O-(4-coumaroyl)-D-quinate 3′-monooxygenase	1.14.14.96	4
CYP84A	ferulate-5-hydroxylase	1.14--	4
COMT	caffeic acid 3-O-methyltransferase	2.1.1.68	12

#### Identification of genes related to flavonoid biosynthesis

**Fig. 11:**
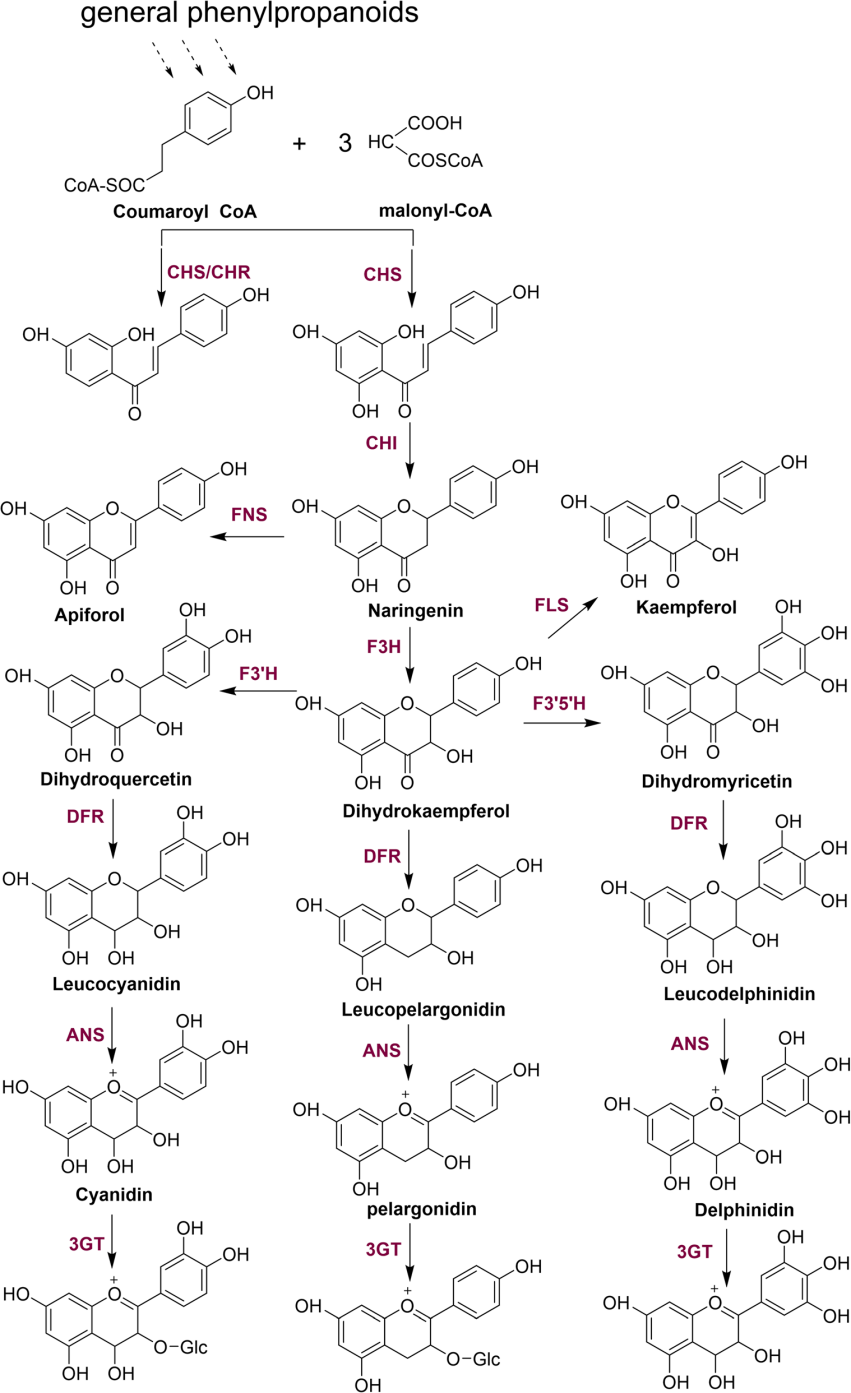
The flavonoid biosynthetic pathway in *R. molle*

Flavonoids are important polyphenolic plant secondary metabolites that can be categorized into flavones, flavonols, flavanone, isoflavones, catechins and chalcones [47]. Appropriate intake of flavonoids can reduce the incidence of cancer, cardio vascular disease, lipid peroxidation and osteoporosis [48]. Previous phytochemical studies have revealed the presence of numerous of flavonoids in flowers of *R. molle* [3, 12, 13]. Considering the diverse beneficial effects of flavonoids, this study also explored the unigenes related to flavonoid biosynthesis in the transcriptome of *R. molle*. Coumaroyl-CoA and malonyl-CoA are the common precursors for the biosynthesis of flavonoids, which are derived from phenylpropanoid pathway and carbohydrate metabolism, respectively. The biosynthesis of flavonoids is initiated by chalcone synthase (CHS), which generates chalcone as the important intermediate and the pathway proceeds with several enzymatic steps for forming other classes of flavonoids, like flavanones and dihydroflavonols. In addition, the side branches of the flavonoid pathway lead to synthesis of other flavonoid classes including isoflavones, flavones, and flavonols (Fig. 11). A total of 53 unigenes related to flavonoid biosynthetic pathway were annotated. Starting from the initial committed enzymes for the biosynthesis of flavonoids, chalcone synthase (CHS), chalcone isomerase (CHI) and flavanone hydroxylase (F3H) were identified, all of which continuously catalyzed p-coumaroyl-CoA and malonyl-CoA into the important intermediate dihydrokae- mpferol. Additionally, the flavonoid-3′-hydroxyase and flavonoid-3′,5′-hydroxyase, which are essential for converting dihydrokaempferol into dihydroquercetin and dihydromyricetin were also identified. The main enzymes involved in flavonoid biosynthesis were listed in Table 5.

**Table 5:** Unigenes involved in the flavonoid biosynthetic pathway in *R. molle*

Enzyme name	Annotation	EC number	Unigene number
CHS	chalcone synthase	2.3.1.74	6
CHI	chalcone isomerase	5.5.1.6	3
F3H	flavanone hydroxylase	1.14.11.9	7
F3’H	flavonoid-3′-hydroxylase	2.7.1.36	2
F3′5’H	flavonoid-3’5’-hydroxylase	1.14.14.81	3
DFR	dihydroflavonol-4-reductase	1.1.1.219	3
ANS	anthocyanidin synthase	1.14.20.4	7
FLS	flavonol synthase	1.14.20.6	2

#### Identification of transcription factors

**Fig. 12:**
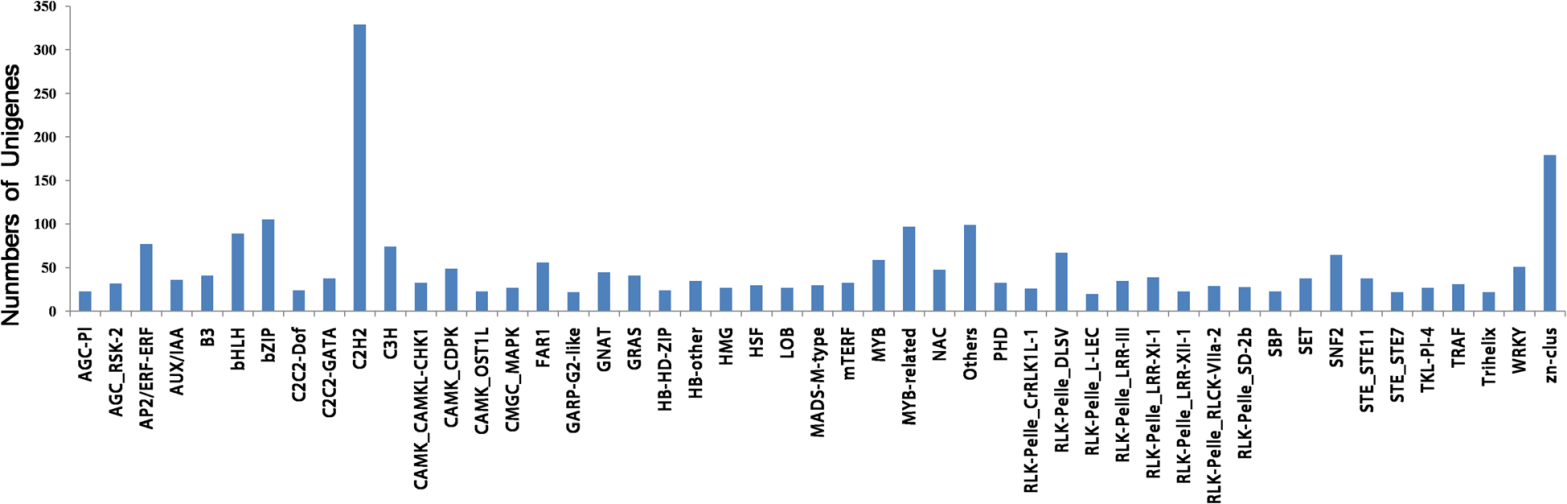
TF family classification of *R. molle* unigenes

In plant, transcription factors (TFs) often play a key role in regulating gene expression at the transcriptional level, which can also affect the metabolic flux by interacting with the promoter regions of gene. Based on our Blast X search against the known Plant Transcription Factor database, 3376 putative *R. molle* transcription factor distributed in at least 49 TF families were identified, which represented 3.35% of the total assembled unigenes (Fig. 12). Among them, C2H2 was the most abundant TF family (329 unigenes, 9.7%), followed by zn-clus (179 unigenes, 5.3%), and bZIP (105 unigenes, 3.1%). C2H2 family members are crucial to plant developmental processes including floral organogenesis, initiation of leaves and lateral shoots and seed development [49]. bZIP regulates processes including pathogen defense and stress signaling [50]. The present study also identified 59 and 97 unigenes encoding MYB and MYB related TFs, respectively. MYB TFs regulate the phenylpropanoid biosynthesis in several plant species and mostly include the R2R3-MYB TFs, which have also been shown to regulate the main branch viz. flavonoid metabolic pathway in phenylpropanoid biosynthesis in several plants including *Arabidopsis thaliana* [51], *Prunus persica* [52] and *Epimedium sagittatum* [52]. In addition, 89, 83 and 51 unigenes were also found to be related to bHLH, AP2-ERF and WRKY, respectively. These TFs have various roles throughout the whole life cycle of plant, from the regulation of several developmental processes to the response to environmental stress [53–55]. Moreover, they are especially important to secondary metabolism in plants. For example, bHLH TFs regulate the flavonoid biosynthetic pathway in plants [56, 57]. The AP2/ERF TF family members modulate the biosynthetic genes for terpenoid indole alkaloids in *Catharanthus roseus* [58]. Further investigation on these TFs may provide a clear profile on the regulatory network for the biosynthesis of secondary metabolites in *R. molle*.

#### Identification of SSRs

Simple sequence repeats (SSRs) also termed as microsatellites, are tandem repeats of short DNA motifs with one to six base pairs. They are widely distributed in eukaryotes (e.g. plants, animals and fungi) as well as in some prokaryotes [59]. SSRs are generally associated with phenotypic variations which have become the most extensively utilized informative molecular markers that favor for a variety of applications, including the genetic breeding of plants, gene mapping and genetic marker-assisted selection [60]. To identify SSRs, all the assembled unigenes of both *R. molle* flower and root were analyzed using MISA. Overall 10,828 SSRs were identified from 7799 unigene, in which the most abundant SSRs were mono-nucleotide repeat motif (4868, 44.95%), followed by di-nucleotide repeat motif (3872, 35.75%) and tri-nucleotide repeat motif (1292, 11.93%), while hexa-nucleotide SSRs (6, 0.055%) had the lowest abundance. Additionally, there were 66 (6.1%) tetra-nucleotide and 14 (0.13%) penta-nucleotide SSR (Additional file 8: Figure S3). Especially, some SSR motifs were associated with the unigenes which encode enzymes involved in terpene biosynthesis (eg. HMGR, DXS) (Table 6). These SSRs can provide a basis for further analyzing genetic diversity of *R. molle* and the related species.

**Table 6:** SSR motifs in unigenes related to terpenoid biosynthesis

Enzyme name	Unigene ID	Number of SSRs	SSR motif	Number of repeats
HMGR	c88044	4	AAC	6
			T	13
			GA	7
			AAC	5
MCT GGPS	c86870	1	TC	7
	c83679	1	*	10
DXS	c83170	1	AG	7
	c84061	1	T	2

* represent compound repeat type. SSR motif: (T) GATCAGCAGAAAGATGAGG ACTTTGATTCATGGTACTGTAACAGCATCTGACGTTTTGCAGG(A)

## Discussion

*R. molle* is a well-known traditional Chinese medicinal plant, which has been extensively investigated on the natural products over the past few years [5–7]. It contains abundant secondary metabolites including terpenoids, flavonoids, and lignans, which account for the broad bioactivities, such as analgesic effect, anti-oxidation and sodium channel modulation [7, 61]. Especially, the grayanane diterpenoids have drawn great attention of researcher due to their special analgesic activity. However, many putative genes involved in the biosynthetic pathways of these complicated compounds, including grayanane diterpenoids, are not clear. Recently, Xiao et al. [21] conducted the transcriptome sequencing analysis of mixed RNA separately extracted from flowers at four developmental stages of *R. molle*. About 20 millions clean reads were generated and assembled into 66,026 unigenes with mean length of 698 bp, among which 31,542 unigenes were annotated in public databases, a total of 55,456 CDSs were also predicted. However, the authors mainly focused on the flowering and flower color formation mechanisms and pay special attention to the key enzymes involved in the carotenoid biosynthetic pathway. In present study, we performed high-throughput transcriptome sequencing for root and flower of *R. molle*. In total, 100,603 unigenes were assembled with the average length of 778 bp and N50 1384 bp, which indicated the assembly integrity was qualified and could be used for subsequent analysis. Among them, 57,416 unigenes were annotated in the public databases. However, 42.9% unigenes remained unannotated, which indicated the published plant transcriptome and genomic data are limited due to few relative species of *R. molle* were sequenced. These unigenes may be related to the biosynthesis of specific secondary metabolites produced by *R. molle*. In addition, there were 76,198 predicted CDSs, accounting for 75.7% of the total unigenes, which are beneficial for further analysis on unigene function at the protein level and facilitate research on pivotal genes. Compared with the study of Xiao et al, our study provided more high-quality clean reads, annotated unigenes and predicted CDSs.

Furthermore, the Gene Ontology assignment program was employed for functional categorization of the annotated unigenes, and a total of 37,108 unigenes obtained GO terms. In a lot of cases, the same unigene was assigned with several terms, thus, 25,634 unigenes were assigned to cellular components, 27,218 to biological processes, and 29,126 to molecular functions. The rest of the unigenes remained unannotated, which may be due to relatively short sequences that were unable to cover the conserved protein domains. Within the molecular function category, the vast majority of unigenes were related to ‘metabolic process’, which were in accordance with the abundant secondary metabolites produced in *R. molle*. Through mapping unigenes onto the KEGG pathways, 232 unigenes were discovered to be enriched in phenylpropanoids biosynthetic pathway, which represented the largest cluster of all secondary metabolic pathways, and this result indicated that the biosynthesis process of phenylpropanoids serves as a reservoir to provide intermediates for the biosynthesis of other diverse secondary metabolites in *R. molle*. Our study also found 53 unigenes related to flavonoid biosynthesis. These annotation and classifications provided a resource for investigating specific pathways in *R. molle*.

Numerous unigenes were involved in terpenoid backbone biosynthesis, including those encoding the well-known enzymes AACT, HMGR, DXR, MCS, IPPI, FPPS and GPPS. Further characterization of other unigenes will improve our understanding of the molecular mechanisms underlying terpenoid biosynthesis. TPS is suggested to be the first committed enzyme in the biological process of terpenoid biosynthesis, which can initially cyclize the common isoprenyl diphosphate precursors (GPP, C-10 FPP, C-15 and GGPP, C-20) to form terpene scaffolds. We mined the transcriptome of *R. molle* flower and root and a panel of TPSs was identified including three diterpene synthase (diTPSs). Out of the three diTPSs, RmTPS6 and RmTPS7 were annotated as copalyl disphosphate synthases and RmTPS8 as kaurene synthase, which were likely related to biosynthesis of kaurene, a presumed biogenetic precursor for grayanane diterpenoids. Further analysis revealed that RmTPS7 expressed only in root, while RmTPS6 and RmTPS8 were more abundant in both the flower and root samples. It implied that RmTPS6 and RmTPS8 are more likely involved in the grayanoid biosynthetic pathway, which is in accordance with the distribution of these terpenoids in both the flower and root samples. The functions of these enzymes need to be further characterized. Moreover, due to the wide distribution of grayanane diterpenoids, it may be difficult to distinguish the differentially expressed biosynthetic gene between the two tissues. Thus, other strategies, including tissue culture combined with methyl jasmonate-induction approach, in combination with multi-level omics may be required to further analyze the biosynthetic pathway of grayanoids.

The structural complexity of grayanoids indicates that most of them carry a high degree of oxygenation, which is quite possible to be catalyzed by CYPs. To explore the molecular underpinnings of terpene backbone modification, 61 unigenes putatively encoding CYPs were identified from the transcriptome, among which four and five unigenes belonged to the CYP71 and CYP76 families, respectively. These two families have been considered as the main driving forces of diterpenoids diversity in plants, which make unigenes from the two families are preference candidates for grayanoid biosynthesis. However, sequence similarity cannot guarantee specific functions due to the functional plasticity within the TPS and CYP families. Therefore biochemical approaches are still necessary for the accurate functional annotation of these TPS and CYP candidates.

In addition, our study examined the differentially expressed unigenes between the flower and root based on FPKM values. The results suggested that several unigenes were uniquely expressed in either the flower or root tissues and many unigenes were expressed at different levels. Further study on these DEGs combined with metabolomes will enable us to more clearly understand the biosynthetic process of secondary metabolites. Besides, transcriptomes also serve as invaluable resource for discovery of SSRs. In the present study, a total of 10,828 SSRs were identified which are slightly more than the SSRs (8266) discovered in Xiao’s work [21]. The distribution and frequency of the classified SSRs between two studies were also different. In our work, the most abundant SSRs were mono-nucleotide repeat motifs (44.95%), followed by di-nucleotide repeat motifs (35.75%). In comparison, Xiao et al reported that di-nucleotide repeat motifs (56.15%) were the most common repeat motifs in the *R. molle* transcriptome [21]. Combination of the two studies will benefit the cultivar fingerprinting, selection of desirable genotypes in *R. molle* breeding and genetic manipulations in *R. molle*. TFs affect the metabolic flux through regulating gene expression. In this work, a total of 3376 TFs were identified, including bHLH (89), AP2/ERF (83), MYB (59), MYB related (97) and WRKY (51) families. The number of TFs in *R. molle* is higher than that of the well-known diterpenoid-producing medicinal plant *S. miltiorrhiza* (1948 TFs), and the model plant *A. thaliana* (2357 TFs), indicating that there are complex metabolic regulation networks in *R. molle*. These TFs may play significant roles in regulating biosynthesis of secondary metabolites. In *Artemisia annua,* the biosynthesis of artemisinin has been effectively regulated by the bHLH transcription factor AabHLH1 [62], in *Catharanthus roseus*, the AP2/ERF members of ORCA2 and ORCA3 can bind to the promoter of the strictosidine synthase (STR) to regulate terpenoid indole alkaloid metabolism [58]. It is of substantial significance to use the genetic engineering methods to control TFs for regulating terpenoid biosynthesis in *R. molle*. Further investigation on these TFs will be helpful for manipulating the metabolic pathways and ultimately increasing the yield of secondary metabolites with medicinal value in *R. molle*.

## Conclusions

In this study, we presented the de novo transcriptome sequencing of *R.molle* flower and root. A total of 100,603 unigenes were generated and 57,416 unigenes were annotated by public databases. Seventeen thousand nine hundred six unigenes were identified by the KEGG database, referring to 133 different plant metabolic pathways. We focused on searching for candidate genes involved in grayanoids biosynthesis, out of 17,906 unigenes, 102 unigenes were involved in terpenoid backbone biosynthesis. Moreover, nine terpene synthases including three diterpene synthase were identified through BLAST similarity search. Sixty-one CYP enzymes were also discovered, among them 9 CYPs were from CYP71 and CYP76 families. The transcriptome information presented in our study also revealed that various genes involved in the biosynthetic pathways of lignans, flavonoids. Additionally, our study identified several transcription factors related to the biosynthesis of secondary metabolites and 10,828 SSRs were also discovered from our transcriptomic database, which are potential for genetic manipulations in *R. molle*. Taken together, the transcriptome data generated in our study, will allow for discovering novel genes involved in specific secondary metabolic pathways, and also provide basis for improving the yields of valuable metabolites in plants or in microbial hosts by metabolic engineering. Moreover, it is also highly valuable to pave the way for functional and comparative genomic studies of this promising medicinal plant in future.

## Supplementary information

**Additional file 1: Table S1.** Accession numbers of protein sequences derived from GenBank and swissprot used for the phylogenetic analysis.

**Additional file 2: Table S2.** TPS and CYP sequences derived from GenBank used for building custom databases.

**Additional file 3: Table S3.** Primers used for qPCR.

**Additional file 4: Figure S1.** (a) Length distribution of assembled unigenes. (b) Length distribution of predict CDSs.

**Additional file 5: Figure S2.** Characterstic of homolgy search of assembled unigenes against NR database. (a) E-value distribution of top Blast hits. (b) Similarity distribution of unigenes. (c) Species distribution of Blast hits.

**Additional file 6: Table S4.** DEGs involved in biosynthetic pathways of secondary metabolites.

**Additional file 7: Table S5.** RNA-Seq data of identified TPS and CYP candidates in *R. molle*.

**Additional file 8: Figure S3.** SSR Density Distribution Map. The X-axis is SSR type, the Y-axis is the number of SSRs per Mb sequence. SSR type (p1: mono-nucleotide repeat motif p2: di-nucleotide repeat motif p3: tri-nucleotide repeat motif p4: tetra-nucleotide repeat motif p5: penta-nucleotide repeat motif p6: hexa-nucleotide repeat motif c: complex repeat motif c*: two SSR sequences with repetitive common parts.

## Data Availability

Raw data of all sample-sequencing results are available at NCBI BioProject database under accession number PRJNA565490 (https://www.ncbi.nlm.nih.gov/bioproject/? term=prjna565490). This Transcriptome Shotgun Assembly project has been deposited at GenBank under the accession GIKT00000000. The version described in this paper is the first version, GIKT01000000 (https://www.ncbi.nlm.nih.gov/ nuccore/GIKT00000000). All the supporting data are included as additional files.

## Abbreviations

AACT: Acetyl-CoA acetyltransferase
CCR: Cinnamoyl-CoA reductase
CDS: Coding sequence
CHI: Chalcone isomerase
CHS: Chalcone synthase
CL: 4-coumarate--CoA ligase
CMS: 2-C-methyl-D-erythritol 4-phosphate cytidylyltransferase
COG: Clusters of Orthologous Groups
CYP: Cytochromes P450
DEGs: Different expressed genes
DMAPP: Dimethylallyl pyrophosphate
DXR: 1-deoxy-D-xylulose-5-phosphate reductoisomerase
DXS: 1-deoxy-D-xylulose-5-phosphate synthase
FPKM: Fragments per kilobase of transcript per million mapped reads
FPP: Farnesyl diphosphate
FPPS: Farnesyl diphosphate synthase
GGPP: Geranylgeranyl diphosphate
GGPPS: Geranylgeranyl diphosphate synthase
GO: Gene Ontology
GPP: Geranyl diphosphate
HDR: 4-hydroxy-3-methylbut-2-enyl diphosphate reductase
HDS: 4-hydroxy-3-methylbut-2-enyl diphosphate synthase
HMGR: Hydroxymethylglutaryl-CoA reductase
HMGS: Hydroxymethylglutaryl-CoA synthase
IPP: Isopentenyl diphosphate
IPPI: Isopentenyl diphosphate isomerase
KEGG: Kyoto Encyclopedia of Genes and Genomes
KOG: EuKaryotic Ortholog Groups
MCS: 2,4-cyclodiphosphate synthase
MEP: Methylerythritol phosphate
MK: Mevalonate kinase
MVA: Mevalonic acid
NJ: Neighbor-Joining
NGS: Next generation sequencing
Nr: NCBI Non-redundant Protein
PAL: Phenylalanine ammonia lyase
Pfam: Protein family
PlnTFDB: Plant transcription factor database
PMK: Phosphomevalonate kinase
TFs: Transcription factors
TPS: Terpene synthases
SSR: Simple sequence repeats
