# Supplementary figures and images for: De novo transcriptome sequencing of Rhododendron molle and identification of genes involved in the biosynthesis of secondary metabolites
Source: BMC Plant Biol. 2020 Sep 4;20:414. doi: 10.1186/s12870-020-02586-y (PMC7487690; doi:10.1186/s12870-020-02586-y)

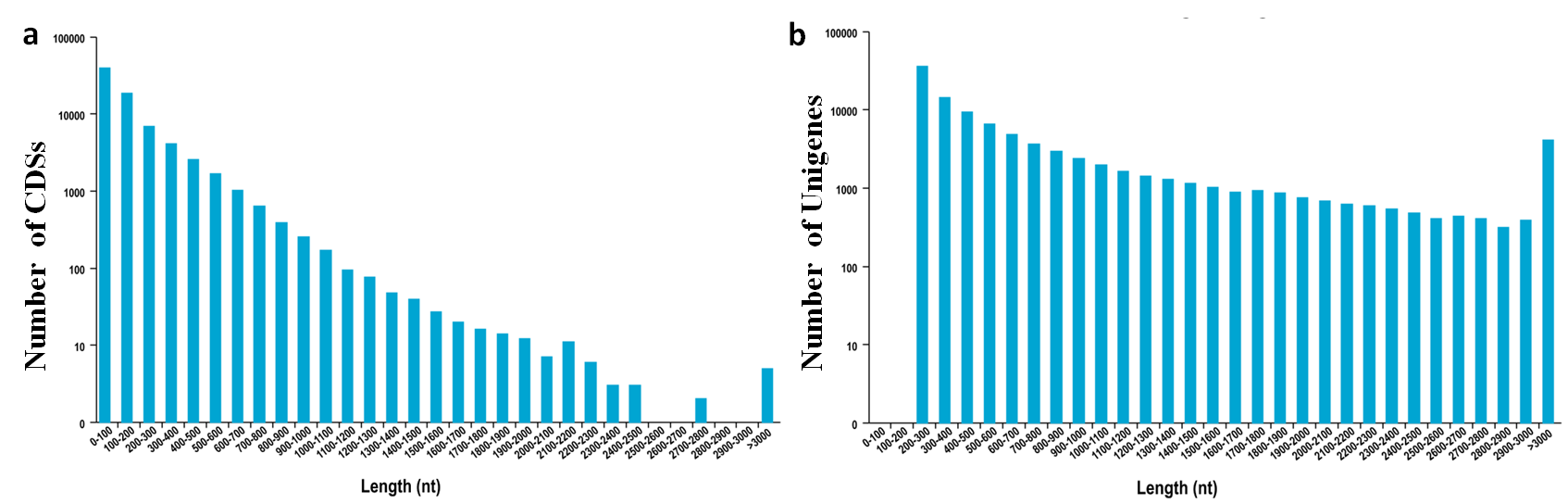

Supplement: Supplementary file 4 — Additional file 4: Figure S1. (a) Length distribution of assembled unigenes. (b) Length distribution of predict CDSs. [file 12870_2020_2586_MOESM4_ESM.tif]

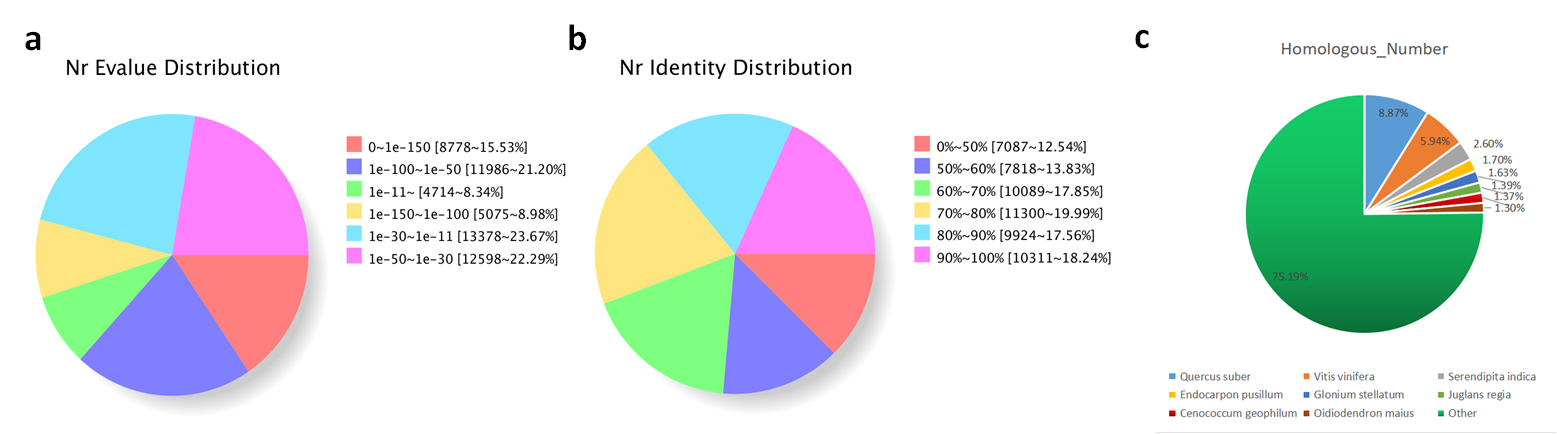

Supplement: Supplementary file 5 — Additional file 5: Figure S2. Characterstic of homolgy search of assembled unigenes against NR database. (a) E-value distribution of top Blast hits. (b) Similarity distribution of unigenes. (c) Species distribution of Blast hits. [file 12870_2020_2586_MOESM5_ESM.tif]

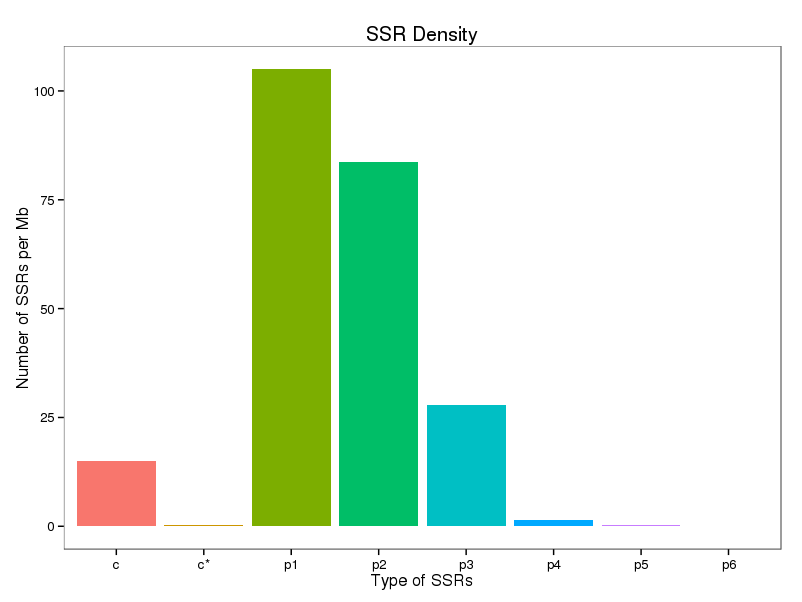

Supplement: Supplementary file 8 — Additional file 8: Figure S3. SSR Density Distribution Map. The X-axis is SSR type, the Y-axis is the number of SSRs per Mb sequence. SSR type (p1: mono-nucleotide repeat motif p2: di-nucleotide repeat motif p3: tri-nucleotide repeat motif p4: tetra-nucleotide repeat motif p5: penta-nucleotide repeat motif p6: hexa-nucleotide repeat motif c: complex repeat motif c*: two SSR sequences with repetitive common parts. [file 12870_2020_2586_MOESM8_ESM.tif]
